# Estimating Latent Distribution of Item Response Theory Using Kernel Density Method

**DOI:** 10.1017/psy.2026.10080

**Published:** 2026-01-08

**Authors:** Seewoo Li, Guemin Lee

**Affiliations:** 1 Department of Education, University of California Los Angeles, USA; 2 Department of Education, Yonsei University, Republic of Korea

**Keywords:** item response theory, kernel density estimation, latent trait distribution, non-normality

## Abstract

The article proposes a new approach to estimating the latent distribution of item response theory (IRT) using kernel density estimation (KDE), particularly the solve-the-equation (STE) algorithm developed by Sheather and Jones (1991). As with existing methods, the KDE method aims to estimate the latent distribution of IRT to reduce biases in parameter estimates when the normality assumption on the latent variable is violated. Simulation studies and an empirical example confirm the robustness of algorithmic convergence of the KDE approach, and show that the KDE approach yields parameter estimates that are more accurate than or comparable to existing methods. Unlike other approaches that require multiple model fits for smoothing parameter selection, KDE requires only a single model-fitting step, substantially reducing computation time. These findings highlight KDE as a practical and efficient method for estimating latent distributions in IRT.

## 1 Introduction

Item response theory (IRT) offers useful psychometric insights from a given item response dataset by modeling the probabilistic relationships between item responses and parameters (van der Linden, 2016). Parametric IRT models postulate ability parameter(s) per examinee and a set of item parameter(s) per item, where the probability of an item response is a function of these parameters. Since biases and errors that occur from the estimation of these parameters can have negative impacts on subsequent decision-making processes, the accuracy of the parameter estimation is crucial.

For the estimation of these parameters, marginal maximum likelihood (MML) estimation has been widely used to separate the ability parameters from the item parameters and construct asymptotically consistent estimators (Baker & Kim, 2004; Glas, 2016). In addition, the MML with the expectation–maximization algorithm (MML-EM; Bock & Aitkin, 1981), which expedites the MML estimation, is widely used for the parameter estimation of IRT models.

Meanwhile, the MML-EM procedure is typically implemented with the normality assumption on the latent distribution (distribution of the latent variable). However, the normality assumption can be violated in practice. For instance, bimodal latent distributions can be observed from the achievement gap within a group of students or from an innate human tendency to be inclined to one side of the latent scale (Harvey & Murry, 1994; Yadin, 2013). Also, skewed latent distributions can be observed by a subgroup that exhibits extreme scale scores or by ceiling/floor effects from the test difficulty relative to the population level in the latent scale (Ho & Yu, 2015; Woods, 2015). The violation of the normality assumption can increase estimation biases, and the estimation of the latent distribution can correct the biases to some extent by reflecting the non-normality (Li, 2024; Woods, 2015).

Some methods have been proposed to estimate the latent distribution to relax the normality assumption, thereby decreasing estimation biases: the empirical histogram method (EHM; Bock & Aitkin, 1981; Mislevy, 1984), the two-component normal mixture model (2NM; Li, 2021; Mislevy, 1984), the Ramsay-curve method (RCM; Woods, 2006), the log-linear smoothing method (LLS; Casabianca & Lewis, 2015), and the Davidian-curve method (DCM; Woods & Lin, 2009).

Meanwhile, the accuracy of density estimation can be further enhanced by adopting asymptotic mean integrated squared error (AMISE) as the loss function. All of the aforementioned methods rely on the likelihood function, which could be attributed to its alignment with the EM algorithm and theoretically ensured convergence (Dempster et al., 1977). This alignment may explain why previous studies have focused almost exclusively on the maximum likelihood estimation (MLE) approach. However, research in density estimation suggests that AMISE is often a more appropriate criterion for achieving accurate density estimates than MLE (Gramacki, 2018; Silverman, 1986; Wand & Jones, 1995). Therefore, rather than adhering strictly to the likelihood function for the sake of EM alignment, adopting AMISE can be considered the preferable choice for improving the accuracy of latent distribution estimation in IRT, unless it undermines the EM convergence.

In the density estimation literature, many studies have focused on the utilization of the kernel density estimation (KDE) method, along with the AMISE criterion, to find the best density estimation method that can be generally applied to various distribution shapes (e.g., Gramacki, 2018; Sheather, 2004; Silverman, 1986; Wand & Jones, 1995). This can be attributed to the broad applicability of KDE and its statistical tractability, especially when the Gaussian kernel is adopted. Up to this point, Sheather and Jones’s (1991) approach has been proven to be the most recommendable method for KDE in unidimensional density estimation (Jones et al., 1996).

This article, thus, aims to propose the application of KDE in the latent distribution estimation of IRT, especially to adopt Sheather and Jones’s (1991) approach. KDE is expected to outperform existing methods on average as it minimizes the AMISE instead of the likelihood, which can be a more suitable loss function for density estimation.

Furthermore, KDE has an advantage over some of the existing methods regarding computation time. Although RCM, LLS, and DCM show an acceptable density estimation accuracy with their hyperparameters controlling the degree of density smoothing, their implementations entail a model selection process, which can be considered as an additional cost for estimating the latent distribution. That is, they need to invest a certain amount of extra time to fit several models according to the hyperparameters that determine the complexity of the distribution, then the best model is determined through a model selection process. In contrast, KDE does not require this model selection process as it has only one density parameter that can be inferred within a single model-fitting procedure. Therefore, KDE can carry out the estimation of latent distribution without necessitating multiple model-fitting procedures, while yielding good performance in estimating the latent distribution.

The objectives of this article are two-fold. First, we propose the approach of applying KDE to latent distribution estimation in IRT. Second, we validate the approach through computer simulations. Simulation studies are particularly valuable because they can uncover characteristics of methods that may be overlooked in theoretical derivations and equations. For example, prior works have shown that the seemingly superior performance of EHM, as reflected in its highest log-likelihood, is in fact the result of overfitting, and that the EM algorithm can fail to converge for semi-nonparametric methods, such as RCM and DCM despite their theoretical guarantees in the EM convergence (Woods, 2006; Woods & Lin, 2009). Thus, simulations aim to evaluate the practical effectiveness of methods, including their stability, accuracy, and computational efficiency, which cannot be directly inferred from theory alone.

The following sections provide mathematical details of the model (Sections 2 and 3), examine its stability, accuracy, and computation time in parameter estimation (Section 4), illustrate its implementation using empirical data (Section 5), and conclude the article with a discussion (Section 6).

## 2 Estimation of latent density

This section aims to explain how the latent density estimation of IRT can be reduced to an ordinary density estimation problem by the EM algorithm of the MML-EM procedure. In particular, it is illustrated through the equations that item parameters and the latent density can be estimated separately, simplifying the problem.

### 2.1 Marginal log-likelihood

Let f(x|θ,τ) be the probability of observed response *x* given ability parameter θ and item parameter vector τ . For example, when the two-parameter logistic model (2PL; Birnbaum, 1968) is used, the function becomes

(1) $$\begin{array}{c} \begin{array}{c} P\\ (\\ \backslash theta\\ ,\\ \backslash boldsymbol\\ \tau \\ )\\ =\\ \backslash Pr\\ (\\ x\\ =\\ 1\\ |\\ \backslash theta\\ ,\\ \backslash boldsymbol\\ \tau \\ )\\ =\\ \backslash frac\\ \backslash exp(a(\theta -b)\\ 1+\backslash exp(a(\theta -b))\\ , \end{array} \end{array}$$

(2) f(x|\theta,\boldsymbolτ)=P^(θ,τ)x^(1−P(θ,τ))1−x,

where x∈{0,1} , $\tau ={\left[a,b\right]}^{'}$ , and *a* and *b* are item discrimination and difficulty parameters, respectively. Then, the likelihood of the *j*th test taker ( j=1,2,⋯,N ) can be expressed as follows:

(3) _Lj(_\thetaj,\codexFontBoldT|\codexFontBold_xj)=_^\prodi=1If(_xji|_θj,_τi),

where *i* denotes items ( i=1,2,⋯,I ), T is item parameter matrix, and $x_{j}={x_{j1},\cdots ,x_{ji},\cdots ,x_{jI}}^{\text{T}}$ is the vector of the *j*th test taker’s item responses.

The MML-EM procedure uses a quadrature scheme to facilitate the EM algorithm. Although other quadrature schemes can be adopted, such as adaptive quadrature (e.g., Haberman, 2006), equidistant points are typically used to approximate the integral. For example, we can focus on the θ range from −6.05 to 6.05 to include the density mass as much as possible when assuming that the mean and standard deviation of the latent density are 0 and 1, respectively. Dividing the range into 121 equal width intervals of 0.1, the quadrature points are then set at the midpoints of these intervals, resulting in points at −6.0,−5.9,−5.8,⋯,6.0 . Using the latent density g(θ,ξ) with the density parameter vector ξ , the marginal log-likelihood of the *N* test takers becomes

(4) logL=logL(T,ξ|X)=loglbrace∏j=1N∫−∞∞Lj(θ,T|xj)g(θ,ξ)dθrbrace=_^∑j=1Nlog∫−∞∞Lj(θ,T|xj)g(θ,ξ)dθ≈_^∑j=1Nlog∑q=1QLj(θq*,T|xj)A(θq*,ξ),

where X is the item response matrix with $X_{j,i}=x_{ji}$ . The subscript *j* on θ is discarded by the independent and identically distributed ( iid ) assumption. Approximating the integral by summation using a quadrature scheme, $\theta_{q}^{*}$ denotes the *q*th quadrature point ( q=1,2,⋯,Q ) and $A(\theta_{q}^{*})=\frac{g(\theta_{q}^{*})}{\sum_{q=1}^{Q}g(\theta_{q}^{*})}$ is a normalized latent distribution at $\theta_{q}^{*}$ . The superscript * is introduced to emphasize the discretization of the continuous variable θ .

### 2.2 Likelihood of latent density

In the expectation step (E-step) of the MML-EM procedure, the quantity $\gamma_{jq}$ is calculated using Bayes’ theorem (Baker & Kim, 2004):

(5) $$\begin{array}{c} \gamma_{jq}={\mathrm{Pr}}_{j}(\theta^{*}=\theta_{q}^{*})=\frac{L_{j}(\theta_{q}^{*},T|X)A(\theta_{q}^{*},\xi )}{\sum_{q=1}^{Q}L_{j}(\theta_{q}^{*},T|X)A(\theta_{q}^{*},\xi )}, \end{array}$$

which is the posterior probability of the *j*th test taker at $\theta^{*}=\theta_{q}^{*}$ .

Using the results from the E-step, the marginal likelihood of the M-step can be expressed as follows:

(6) $$\begin{array}{ccccc} \log L(T,\xi |X) & \approx \sum_{j=1}^{N}\log\left[\sum_{q=1}^{Q}L_{j}(\theta_{q}^{*},T|X)A(\theta_{q}^{*},\xi )\right] & =\sum_{j=1}^{N}\sum_{q=1}^{Q}\gamma_{jq}\log\left[L_{j}(\theta_{q}^{*},T|X)A(\theta_{q}^{*},\xi )\right]-\sum_{j=1}^{N}\sum_{q=1}^{Q}\gamma_{jq}\log\gamma_{jq} & =\sum_{j=1}^{N}\sum_{q=1}^{Q}\gamma_{jq}\log\left[L_{j}(\theta_{q}^{*},T|X)\right]+\sum_{j=1}^{N}\sum_{q=1}^{Q}\gamma_{jq}\log\left[A(\theta_{q}^{*},\xi )\right]-\sum_{j=1}^{N}\sum_{q=1}^{Q}\gamma_{jq}\log\gamma_{jq} & =\;\ell_{item}\;+\;\ell_{density}\;-\;\text{constant}. \end{array}$$

The separation of the likelihood demonstrates the statistical independence of the item parameters T and density parameters ξ . Consequently, the density parameters depend only on $\ell_{density}$ .

### 2.3 Existing methods

Several latent density estimation methods have been proposed to flexibly model the distribution of latent traits in IRT. These methods vary in how they represent the latent distribution, ranging from simple empirical estimates to flexible nonparametric approaches. While these approaches aim to relax the normality assumption underlying the MML estimation, they differ in estimation accuracy, interpretability, computational burden, and availability of model evaluation criteria.

EHM (Bock & Aitkin, 1981; Mislevy, 1984) is a nonparametric approach that directly utilizes the expected frequencies calculated in the E-step of the EM algorithm. Specifically, it estimates the latent density at each quadrature point as the normalized sum of posterior probabilities across respondents. This method is computationally efficient and easy to implement, since results from the E-step can be directly transformed into a density estimate without additional computation. However, because it does not apply any smoothing, EHM is sensitive to sampling noise, which can result in erratic density shapes and suboptimal item parameter estimates (Li, 2021; Woods, 2015; Woods & Lin, 2009).

DCM (Woods & Lin, 2009) and RCM (Woods, 2006) both aim to improve upon EHM by smoothing the noisy expected frequencies. These methods differ in the functional forms they employ for density estimation, but share a common estimation strategy and model selection procedure. DCM models the latent distribution as a squared polynomial multiplied by a standard normal density. The order of the polynomial, denoted by *h*, determines the complexity of the distribution: when h=1 , the model reduces to the normal distribution, while larger values of *h* allow for increasingly flexible density shapes. To ensure that the resulting function is a proper probability distribution, the polynomial coefficients are constrained via a polar coordinate transformation. On the other hand, RCM uses basis splines, where the order of the splines and the number of breaks are the hyperparameters that control the smoothness of the density. In the estimation of density parameters of RCM, it employs a normal prior to improve estimation stability. However, the standard deviation of the prior is often chosen through trial and error. Both methods require the selection of smoothing parameter(s), which is typically achieved by fitting multiple models and selecting the best one based on the Hannan–Quinn (HQ) criterion (Hannan & Quinn, 1979). This criterion, which is similar to information criteria, such as the Akaike information criterion (AIC) and the Bayesian information criterion (BIC), balances model flexibility and overfitting. For RCM, however, visual inspection of the candidate models is also necessary in addition to the HQ criterion.

LLS (Casabianca & Lewis, 2015) is another approach that aims to regularize the noisy frequency estimates from EHM by fitting a log-linear model to the expected posterior counts. It expands the log-density as a linear combination of polynomials, and its hyperparameter controls the smoothness of the density by determining the highest degree of the polynomials. However, unlike DCM, the model selection strategy of LLS has not been clearly established to determine the optimal hyperparameter.

The 2NM (Li, 2021; Mislevy, 1984) is a parametric alternative that extends the standard normality assumption by modeling the latent distribution as a weighted sum of two normal distributions. This added flexibility allows 2NM to capture deviations from normality, such as skewness or bimodality. The density parameters are estimated using a secondary EM algorithm nested within the MML-EM framework. While the parametric form of 2NM offers advantages, such as interpretability and analytic simplicity, it also imposes constraints on flexibility, making it less capable of capturing complex or highly irregular latent distributions compared to nonparametric or semi-nonparametric approaches.

## 3 Kernel density estimation in IRT

### 3.1 Kernel density estimation

KDE is a nonparametric density estimation method that assigns a kernel function to each observed data point (Silverman, 1986). Let the vector $z={\left[Z_{1},Z_{2},\cdots ,Z_{N}\right]}^{\text{T}}$ denote *N* observed data points. The kernel functions formulate the density as follows:

(7) $$\begin{array}{c} g(z|h)=\frac{1}{Nh}\sum_{j=1}^{N}K\left(\frac{z-Z_{j}}{h}\right), \end{array}$$

where K(⋅) is the kernel function and *h* is the hyperparameter called *bandwidth*. The bandwidth controls the degree of density smoothing by determining the width of the kernel function. For example, when the Gaussian kernel is applied, the bandwidth *h* represents the standard deviations of the kernels. Among the available kernel functions, such as Epanechnikov, biweight, triangular, Gaussian, and uniform, the Gaussian kernel is most commonly adopted due to the minimal influence of kernel choice on the resulting density estimates and the differentiability of the Gaussian form (Gramacki, 2018; Silverman, 1986). Following this convention, the present study focuses on the Gaussian kernel and its applications.

**Figure 1:**
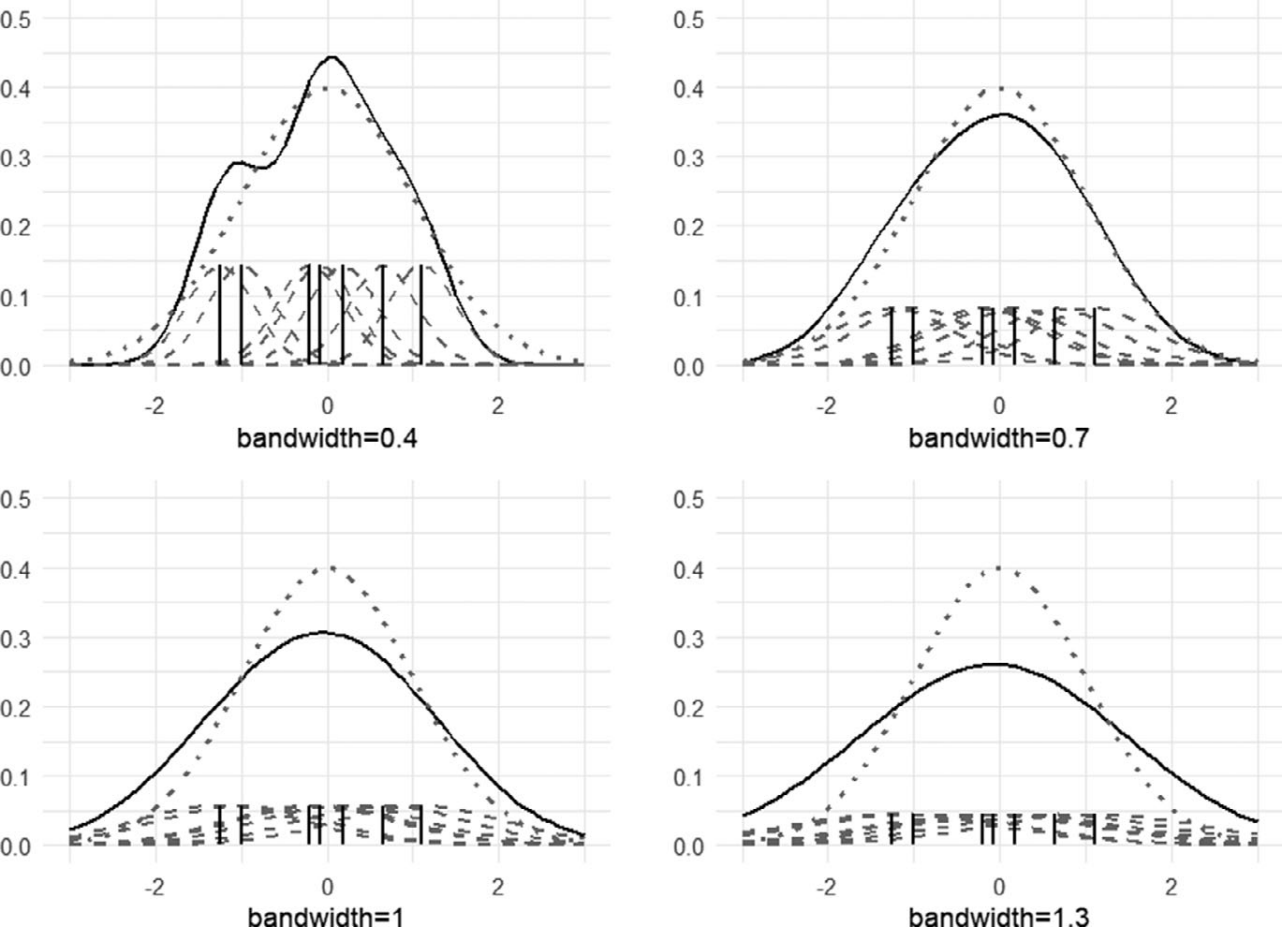
Estimated densities according to different *h* values. Figure 1 long description.

In Figure 1, for illustrative purposes, the standard normal distribution (dotted lines) is estimated using four different bandwidth values (i.e., 0.4, 0.7, 1.0, and 1.3). Using the seven data points generated from the standard normal distribution, seven kernel functions (dashed lines) are formulated. Mathematically, kernel functions are Gaussian distributions with their means as the seven data points and a common standard deviation of *h*, and are scaled by the total sample size: $\frac{1}{N}=\frac{1}{7}$ . The estimated densities (solid lines) are summations of the kernel functions. Among the four density estimates, the standard normal distribution is best recovered when h=0.7 . In comparison, the function is under-smoothed when h=0.4 , resulting in a bimodal distribution. When h=1.0 and h=1.3 , the flatter shapes compared to the standard normal distribution indicate that they were over-smoothed. Considering that the optimal bandwidth is h=0.7177 for this example, it is shown that the density estimate is most accurate when the bandwidth *h* was closest to the optimal value (see Gramacki, 2018 for the formula for calculating the optimal bandwidth when the true density is known).

Importantly, the bandwidth *h* of KDE determines the degree of smoothing, without necessitating an estimation of additional distribution parameters. This characteristic of KDE obviates the need to include multiple model-fitting procedures and a model selection process to accurately estimate the latent density.

### 3.2 Bandwidth estimation and approximate mean integrated square error (AMISE)

As bandwidth estimation (or optimal bandwidth selection) itself becomes the density estimation of KDE, the search for an optimal bandwidth is crucial to obtain accurate density estimates. To this end, existing literature on density estimation has focused on the AMISE criterion for bandwidth estimation (see Gramacki, 2018; Silverman, 1986; Wand & Jones, 1995). Consequently, within this context, the optimal bandwidth can be defined as the one that minimizes the AMISE criterion.

AMISE is the approximation of the mean integrated squared error (MISE) obtained from a Taylor expansion (Jones, 1991; Sheather, 2004; Silverman, 1986; Wand & Jones, 1995), where $\text{MISE}(\hat{g})=E\left[\int \left.l,b,r,a,ce\hat{g}(\theta )-g(\theta ),r,b,r,a\right.ce^{2}dx\right]$ is an appropriate index for evaluating the density estimation results (Jones, 1991). AMISE can be expressed as

(8) $$\begin{array}{c} \text{AMISE}(\hat{g_{h}})=\frac{1}{Nh}R(K)+\frac{h^{4}}{4}\mu_{2}{\left(K\right)}^{2}R(g"), \end{array}$$

where $\hat{g_{h}}$ is the density estimate using the bandwidth *h*, $R(K)=\int K{\left(t\right)}^{2}dt$ , $\mu_{2}(K)=\int t^{2}K(t)dt$ , and $R(g")=\int g"{\left(t\right)}^{2}dt$ . When the Gaussian kernel is used for the kernel *K*, it can be obtained from a simple algebra that $R(K)={\left(2\sqrt{\pi }\right)}^{-1}$ and $\mu_{2}(K)=1$ .

Another equation can be obtained by differentiating Equation (8) with respect to *h* and equating it to 0 :

(9) $$\begin{array}{c} \hat{h_{\text{AMISE}}}={\left[\frac{R(K)}{N\mu_{2}{\left(K\right)}^{2}\psi_{4}}\right]}^{\frac{1}{5}}, \end{array}$$

where $\hat{h_{\text{AMISE}}}$ denotes the bandwidth estimate from the AMISE criterion, $\psi_{d}=E_{t}\left[g^{\left(d\right)}(t)\right]$ , and $\psi_{4}=\int g^{\left(4\right)}(t)g(t)dt=\int g"{\left(t\right)}^{2}dt=R(g")$ . Unfortunately, Equation (9) is not a closed-form solution to estimate *h* as it depends on the information from an unknown density *g*, which is $\psi_{4}=R(g")$ . Therefore, one of the widely used strategies is to calculate the estimate $\hat{\psi_{4}}$ and plug it into Equation (9), and consequently, the accurate estimation of $\psi_{4}$ determines the overall performance of KDE (Gramacki, 2018; Sheather, 2004; Sheather & Jones, 1991; Wand & Jones, 1995).

### 3.3 Plug-in method

Among the three types of bandwidth selection methods—*rule-of-thumb*, *cross-validation*, and *plug-in*—the plug-in method is known to produce a bandwidth estimator that has an efficient convergence rate to the parameter and small bias and variance (Gramacki, 2018; Harpole et al., 2014; Sheather, 2004; Sheather & Jones, 1991; Wand & Jones, 1995). Due to its precision and efficiency in bandwidth estimation, the plug-in method has been a recommended option for density estimation in statistical software programs, such as R and SAS (R Core Team, 2025; SAS Institute Inc., 2020).

*Direct plug-in* (DPI) and *solve-the-equation* (STE) are two types of plug-in methods. Within the overall scheme of the $\psi_{4}$ estimation, both plug-in methods utilize $\psi_{d+2}$ in estimating $\psi_{d}$ , where an even number *d* is the order of differentiation and $p_{d}$ is a pilot bandwidth introduced to estimate $\psi_{d}=E\left(g^{\left(d\right)}(x)\right)$ (Gramacki, 2018; Sheather, 2004; Wand & Jones, 1995). That is, we start the algorithm by setting *d* to a specific value (e.g., d=8 ), then iteratively estimate $\psi_{d-2}$ using $\psi_{d}$ until we get the estimate of $\psi_{4}$ . In this study, we focus on STE (Sheather & Jones, 1991) between the two, which is the most widely used method in unidimensional density estimation due to its small bias and variance in bandwidth estimation (Gramacki, 2018; Harpole et al., 2014; Jones et al., 1996; Sheather, 2004; Sheather & Jones, 1991). STE is a suggested option in R (R Core Team, 2025) and is set as the default in SAS (SAS Institute Inc., 2020). Note that, at the time of writing this article, STE is not fully extended to multidimensional cases. In comparison, DPI showed more accurate and stable performance in estimating the multidimensional bandwidth compared to rule-of-thumb and cross-validation (Chacón & Duong, 2010; Duong & Hazelton, 2003).

To implement STE, some additional equations are used:

(10) $$\begin{array}{c} p_{d}={\left[\frac{2K^{\left(d\right)}(0)}{-\mu_{2}(K)\psi_{d+2}(p_{d+2})N}\right]}^{\frac{1}{d+3}}, \end{array}$$

(11) $$\begin{array}{c} \psi_{d}^{N}={\left[\frac{{\left(-1\right)}^{d/2}d!}{{\left(2\hat{\sigma }\right)}^{d+1}\left(\frac{d}{2}\right)!\pi^{1/2}}\right]}^{1/9}, \end{array}$$

and

(12) $$\begin{array}{c} p(h)={\left[\frac{2K^{\left(4\right)}(0)\mu_{2}(K)\hat{\psi_{4}}(p_{4})}{-\hat{\psi_{6}}(p_{6})R(K)}\right]}^{1/7}h^{5/7}. \end{array}$$

Above, Equation (10) provides the unbiased estimator of $p_{d}$ using the information of $\psi_{d+2}$ , Equation (11) is the formula to compute $\psi_{d}$ assuming that the distribution is normal, and Equation (12) expresses a pilot bandwidth $p_{4}$ as a function of *h* (Gramacki, 2018; Sheather & Jones, 1991).

The algorithm can be summarized as follows (Gramacki, 2018):

1. Calculate $\psi_{6}^{N}$ and $\psi_{8}^{N}$ from Equation (11).
2. Calculate $p_{4}$ and $p_{6}$ from Equation (10) using $\psi_{6}^{N}$ and $\psi_{8}^{N}$ .
3. Estimate $\psi_{4}(p_{4})$ and $\psi_{6}(p_{6})$ using $p_{4}$ and $p_{6}$ , respectively.
4. Estimate $\psi_{4}$ using the estimator $\hat{\psi }\left(p(h)\right)$ , where p(h) is calculated from Equation (12) using $\hat{\psi_{4}}(p_{4})$ and $\hat{\psi_{6}}(p_{6})$ obtained from *Step 3*.
5. The estimated bandwidth $\hat{h}$ is the numerical solution of Equation (9) when $\hat{\psi }\left(p(h)\right)$ is plugged into the equation.

Note that the illustrated algorithm above is the two-step algorithm where “two-step” refers to the process of starting from d=8 and terminating at d=4 . In the same way, the three-step algorithm starts from d=10 , then sequentially moves to 8 , 6 , and 4 . Although increasing the number of steps reduces bias, the two-step algorithm is generally preferred due to the trade-off between bias and variance.

### 3.4 Application of KDE to IRT

As with other IRT latent density estimation methods, KDE can be applied to the latent density estimation of IRT by treating $\hat{f_{q}}=\sum_{j=1}^{N}\gamma_{jq}$ as observed data points, where $\hat{f_{q}}$ is the expected frequency of test takers at $\theta^{*}=\theta_{q}^{*}$ . For the ease of programming, $\hat{f}$ is rounded in this study. Suppose, for illustration, that we are in the *i*th iteration of the MML-EM procedure, where “*i*” temporarily denotes the sequence of the EM iteration, not related to the subscript *i* used for items. Using $\hat{f_{q}}$ obtained from the E-step of the *i*th EM iteration, the STE algorithm in Section 3.3 is implemented to get $\hat{h}$ during the M-step of the *i*th EM iteration. The density estimate $g(\theta |\hat{h})$ (see Equation (7)) is first scaled to have a mean of 0 and a standard deviation of 1 using linear interpolation and extrapolation, which is also applied to DCM to identify the latent scale (Woods & Lin, 2009). Then, it becomes the estimated latent distribution of the *i*th EM iteration, which, in turn, is used in the E-step of the (i+1) th EM iteration. When the MML-EM procedure converges, $g(\theta |\hat{h})$ from the last EM iteration is taken as the estimated latent distribution.

Unlike other methods that employ additional density parameters according to the hyperparameter *h*, only the bandwidth *h* is involved during the application of KDE. As a result, KDE can yield a smoothed latent density estimate within a single MML-EM procedure.

## 4 Simulation study

### 4.1 Compared methods

To evaluate the accuracy of the KDE method in parameter estimation using computer simulation techniques, the performance of KDE is assessed under various simulation conditions by comparing it with three existing approaches: the normality-assumption method (NM), EHM, and DCM.

NM refers to the conventional approach that does not estimate the latent distribution but instead assumes normality. It serves as a baseline model—similar to a null model—against which the advantages of more flexible approaches can be evaluated. In a strict sense, NM differs from the other approaches because it does not attempt to estimate the latent density.

EHM provides a baseline among density estimation methods, as it relies on normalized histograms without smoothing. DCM further smooths the non-smoothed density estimate produced by EHM at every EM iteration. DCM has shown competitive accuracy among existing methods and can offer a meaningful benchmark for assessing new techniques (Woods & Lin, 2009). In addition, its principled model selection procedure based on the HQ criterion makes it particularly suitable for fair comparisons.

Other methods, such as RCM, LLS, and 2NM, are omitted from the comparison due to challenges in evaluating their performance on equal grounds. RCM and LLS both involve subjective decisions in the model selection process: RCM requires trial and error in setting the standard deviation of the prior distribution and often relies on visual inspection, while LLS lacks a clear model selection guideline. The 2NM method depends heavily on the choice of the true density in the simulation design due to its parametric nature. This dependency is misaligned with the focus of the present study on density estimation methods applicable when little or no information about the true density is available, which is the motivation for adopting nonparametric approaches.

Accordingly, NM, EHM, and DCM are chosen to represent the null, lower, and upper bounds of existing approaches in terms of flexibility and performance.

### 4.2 Simulation design

**Figure 2:**
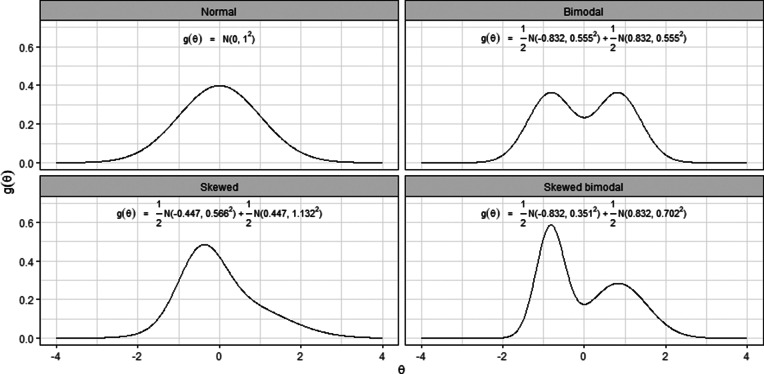
Normal, skewed, bimodal, and skewed bimodal distributions used for the simulation study. Figure 2 long description.

The simulation conditions are formulated by three factors that can potentially affect the results: the shape of the latent distribution, the length of the tests, and the number of test takers. Figure 2 shows the four shapes of the latent distribution used for the simulation study: normal, skewed, bimodal, and skewed bimodal. The three non-normal distributions are formulated by a mixture of two normal components, and their mean and variance are 0 and 1 , respectively (see Li, 2021 for reparameterization of the two-component normal mixture distribution to fix the overall mean and variance). Setting the standard normal distribution as a reference, the skewed and bimodal distributions represent the two types of normality violation, and the skewed bimodal distribution represents the combination of the two sources of violation. The skewness values of the normal and bimodal distributions are both 0. In contrast, the skewed and skewed bimodal distributions have skewness values of approximately 0.6 and 0.5, respectively. These values were chosen to approximate the skewness observed in the IRT scale score distributions of the U.S. state-level testing programs (Ho & Yu, 2015). For example, the median skewness of the 24 IRT scale score distributions in Colorado was approximately −0.6 , whereas in New York, it was approximately 0.7 , ignoring the sign of skewness. The shape of the skewed bimodal distribution is designed to resemble the example of bimodal test score distributions presented in Sibbald (2014). Although a symmetric bimodal distribution may not frequently occur in practice as a latent distribution, it is included in the simulation conditions to examine the effect of bimodality independently of skewness. Test lengths of 8, 15, 30, and 60 items can be viewed as representing very short, short, medium, and long tests, respectively. Very short or short tests include brief scales, such as the 8-item version of the Center for Epidemiologic Studies Depression (CES-D8) scale (Missinne et al., 2014) and end-of-chapter quizzes in online learning platforms, where the latent distribution has a relatively strong influence on parameter estimation. Examples of long tests are large-scale educational assessments, such as the Scholastic Aptitude Test and the American College Testing. Lastly, the number of test takers is varied—set at 500, 1,000, and 2,000—to examine the performance of the methods with respect to sample size.

For each of the 48 simulation conditions (4 distributions × 4 test lengths × 3 sample sizes), 200 item response datasets are generated using the 2PL (Birnbaum, 1968) and fitted by NM, EHM, DCM, and KDE. For the random sampling of item parameters, the *a* parameter is generated from a uniform distribution from 0.8 to 2.5 , and the *b* parameter is generated from the standard normal distribution truncated at ±2 . These parameters serve as true values in calculating the evaluation criteria in Equations (14) and (15). The IRTest package (Li, 2024; R Core Team, 2025)—an R package specialized in latent density estimation of IRT—is used for both data generation and model fitting. The source code for the simulation study is available at https://github.com/SeewooLi/KDE_IRT_code. The KDE option in IRTest has been developed particularly for this study. An MML-EM procedure is considered to be successfully converged when the maximum parameter change drops below 0.0001 within the specified maximum number of EM iterations. The maximum number of iterations is set to 200 for NM, DCM, and KDE, and to 2,000 for EHM to accommodate its slower convergence. These settings are consistent with previous studies that used similar criteria to determine convergence of the MML-EM procedure (Woods, 2006; Woods & Lin, 2009). An AMD Ryzen 7 5700G processor is used for the study.

The accuracy of the density estimation is evaluated by the integrated squared error (ISE). In particular, ISE from NM can indicate the degree to which the true density deviates from the normal distribution, because NM assumes the latent distribution to be normal and, thus, does not estimate it. The ISE criterion can be written as follows:

(13) \textISE(g^)=_^∫−∞∞g^(θ)−g(θ)2dθ≈_^∑q=1121110g^(θq*)−g(θq*)2.

The integration is approximated by the quadrature scheme of using 121 equally spaced grids from −6 to 6.

Lastly, the root mean square error (RMSE) evaluates the accuracy of the estimation of the ability parameter θ , item characteristic curve (ICC), and the item parameters (*a* and *b*):

(14) \textRMSE(\hat\textICC)=\sqrt\frac1I_^∑i=1I∑q=1121Pi^(θq*)−Pi(θq*)2A^(θq*),

(15) \textRMSE(\hatτ)=\sqrt\frac1I_^∑i=1Iτ^−τ2,

and

(16) \textRMSE(\hatθ)=\sqrt\frac1N_^∑i=1Nθ^−θ2,

where $\hat{P_{i}}(\theta_{q}^{*})$ in Equation (1) is the estimated ICC of Item *i* evaluated at $\theta_{q}^{*}$ , $\hat{A}(\theta_{q}^{*})$ is the estimated normalized density (see Section 2.1) at the *q*th grid, and τ represents either the *a* or *b* parameter. Expected *a priori* (EAP) scores are used for $\hat{\theta }$ . Based on 200 data replications, the average RMSE and ISE values for each method are reported in Section 4.3.

### 4.3 Results

The MML-EM procedure successfully converged for both the NM and KDE models across all datasets (i.e., 200 replications for each of the 48 simulation conditions) within 200 EM iterations, using a convergence threshold of 0.0001. In contrast, 2.51% (241 out of 9,600) of the “best” DCMs and 8.53% (8,184 out of 96,000) of all DCMs failed to converge successfully. For EHM, the MML-EM procedure did not converge within 2,000 EM iterations in 8.92% of cases (856 out of 9,600), where many of the non-converged cases occurred when the number of items was 8 (499 cases). The average computation times for model fitting were 4.86 seconds for NM, 17.74 seconds for EHM, 78.76 seconds for DCM, and 4.26 seconds for KDE. The relatively longer time of EHM is mainly due to its slow convergence that required a large number of iterations for model fitting, and the substantially longer duration observed for DCM is largely due to the need to fit 10 models. Interestingly, the average elapsed time for NM exceeded that of KDE, despite KDE involving an additional step for bandwidth estimation. Two factors may explain this finding. First, computation in the E-step dominates the overall MML-EM procedure, as it involves evaluating probabilities for every combination of item, person, and latent grid point. Thus, the additional cost of the bandwidth estimation in KDE is comparatively negligible. Second, density estimation in KDE may have facilitated faster identification of a local maximum on the likelihood surface compared to NM, requiring 44.5 iterations for convergence in KDE versus 49.5 for NM.

**Figure 3:**
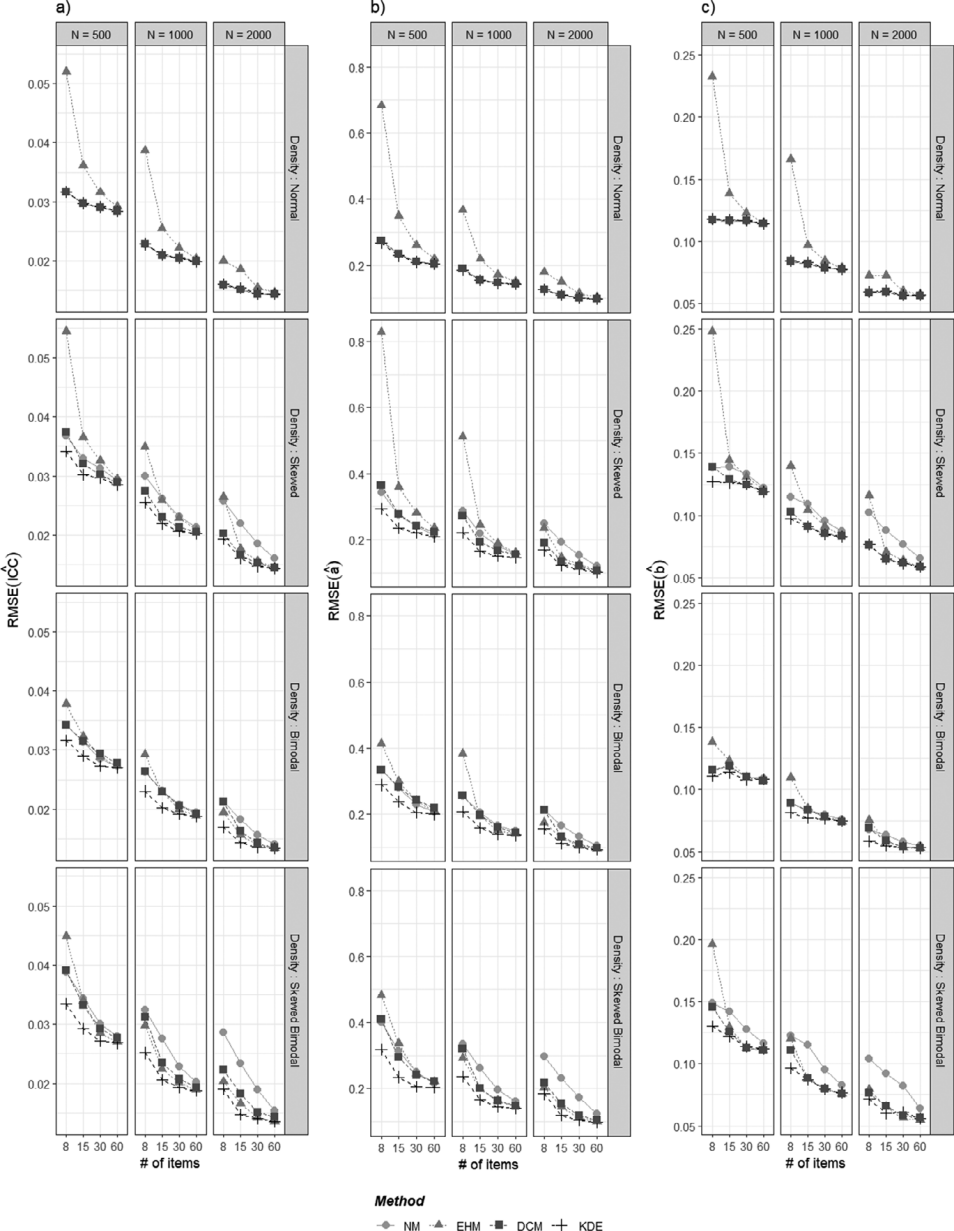
Simulation results of $RMSE(\hat{ICC})$ , $RMSE(\hat{a})$ , and $RMSE(\hat{b})$ . *Note*: Panels (a)–(c) present RMSE values for ICC, *a*, and *b*, respectively. Figure 3 long description.

**Figure 4:**
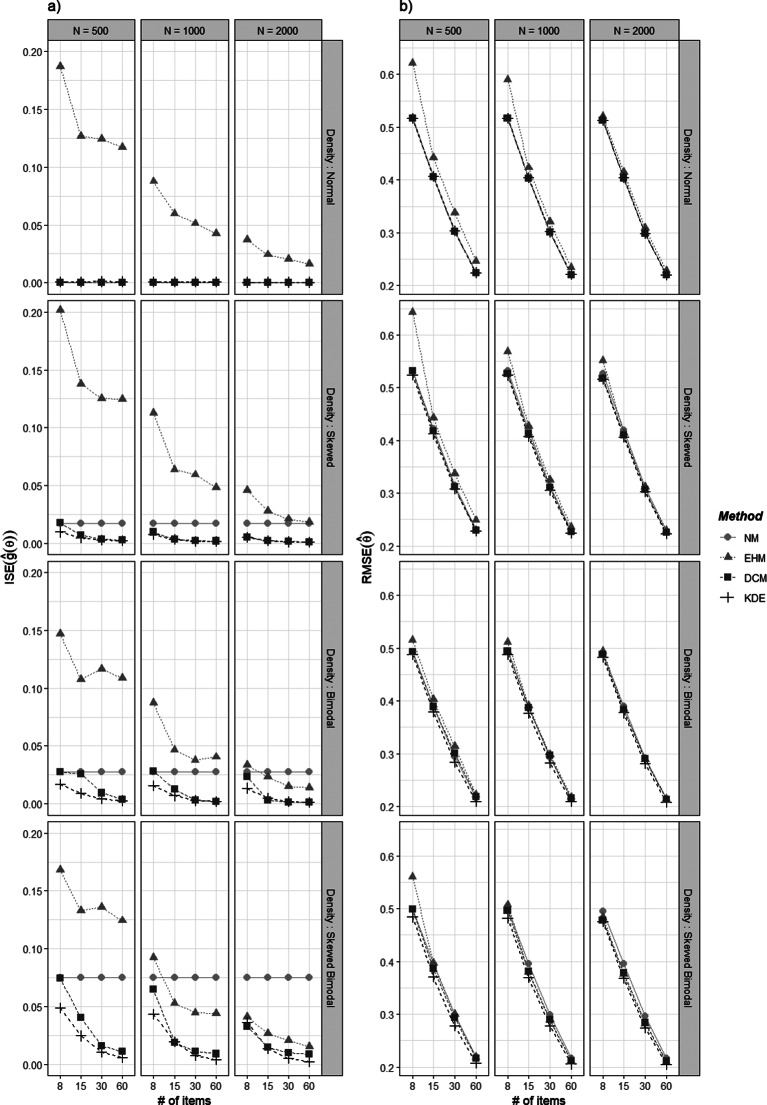
Simulation results of $ISE(\hat{g}(\theta ))$ and $RMSE(\hat{\theta })$ . *Note*: Panels (a) and (b) present $ISE(\hat{g}(\theta ))$ and $RMSE(\hat{\theta })$ , respectively. Figure 4 long description.

Figures 3 and 4 summarize the simulation results using the RMSE and ISE criteria, where lower values indicate greater estimation accuracy. Tables A1 and A2 in the Appendix report the calculated RMSE and ISE values. As illustrated in Figure 3, RMSE values generally decreased as both the sample size and the number of items increased. Under the normal latent distribution, NM, DCM, and KDE exhibited nearly identical RMSE values, whereas EHM produced larger RMSE values unless the number of items was sufficiently large. Under non-normal latent distributions, DCM and KDE achieved lower RMSE values than NM. The performance of EHM in these non-normal conditions varied depending on the simulation design: it effectively reduced RMSE relative to NM when both the sample size and the number of items were adequate, but yielded higher RMSE otherwise. Notably, although there are some cases in which EHM or DCM slightly outperformed KDE, KDE consistently produced the smallest or comparable RMSE values across all conditions, indicating its robust accuracy. The advantage of KDE, relative to the other methods, was particularly pronounced when the sample size and the number of items were limited.

The ISE values are presented in Figure 4a. Consistent with the item-level results (i.e., ICC and the *a* and *b* parameters), the accuracy of latent density estimation generally improved with larger sample sizes and greater numbers of items for EHM, DCM, and KDE. The ISE values of EHM often exceeded those of NM due to overfitting. In comparison, both DCM and KDE accurately recovered the normal latent distribution and substantially reduced ISE under non-normal conditions in most cases. However, the recovery of the latent distribution by DCM was limited when both the sample size and the number of items were small. KDE achieved the lowest or nearly lowest ISE values across all conditions, with the one exception (skewed bimodal distribution, 8 items, and 500 examinees) where DCM slightly outperformed KDE. These findings could explain the accuracy of KDE in item parameter estimation, as accurate identification of the latent distribution influences the precision of the item parameter estimates.

In parallel, Figure 4b shows that KDE also provided an accurate recovery of the ability parameters, with $RMSE(\hat{\theta })$ values that were the lowest or comparable to those of EHM and DCM across the simulation conditions. Although the enhanced accuracy of KDE in ability estimation may be practically meaningful in contexts such as high-stakes testing, the results in Panel (b) indicate that the number of items exerted the greatest influence on the accuracy of ability parameter estimates.

## 5 Empirical illustration

The effectiveness of KDE can be illustrated using real data. For this purpose, the CES-D8 scale (e.g., Missinne et al., 2014; Radloff, 1977) is employed. The scale measures depressive symptomatology in the general population through binary items asking the presence of symptoms associated with depression. The CES-D8 was administered in the COVID-19 Coping Study (Kobayashi & Finlay, 2022), which is publicly available at https://www.openicpsr.org/openicpsr/project/131022/version/V3/view. The source code for the following analyses is available at https://github.com/SeewooLi/KDE_IRT_code. For illustration, we use item responses from 2,372 respondents collected during the 6th month of the study.

Following the estimation procedures outlined in Section 4, the MML-EM algorithm successfully converged for all models considered (NM, EHM, DCM, and KDE). Based on the HQ criterion (Hannan & Quinn, 1979), the DCM with a hyperparameter of h=3 was selected as optimal.

**Figure 5:**
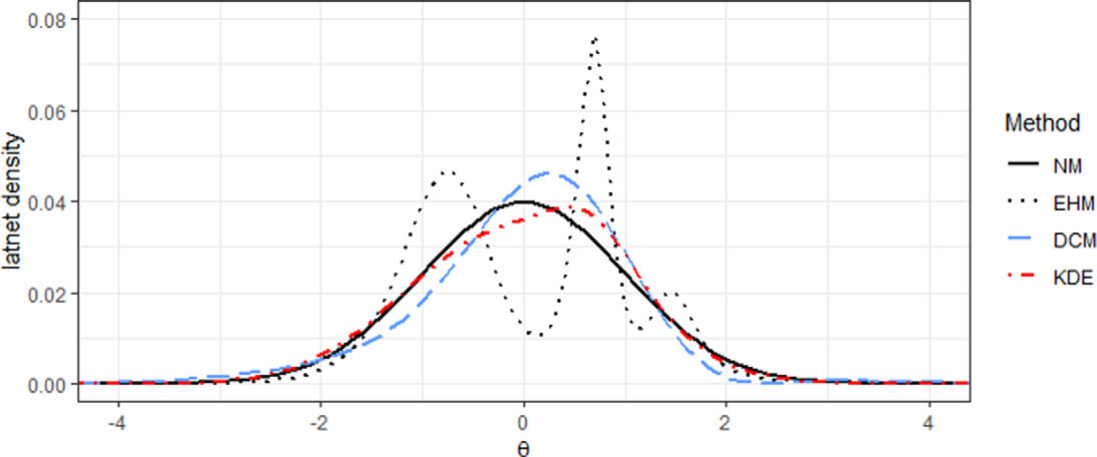
The estimated latent densities from EHM, DCM, and KDE for the CES-D8 data. *Note*: The standard normal distribution represents the latent distribution of NM, which is typically not estimated. Figure 5 long description.

Figure 5 shows the estimated densities from EHM, DCM, and KDE. All methods indicate moderate left skewness, suggesting the existence of a small subgroup with distinct response patterns. However, the density shapes vary: both DCM and KDE produce smooth estimates, while EHM yields a jagged distribution that may not accurately reflect the true latent distribution.

To quantify the effectiveness of the density estimation methods, summed score likelihood-based statistics (Li & Cai, 2012, 2018) can be used:

(17) \bar^X2=N_^\sums=0S−1\frac^(ps−πs^)2_^πs,

where S=9=1+8 represents the total number of summed scores, including the summed score of 0. Here, $p_{s}$ denotes the observed proportion of summed score *s*, and $\hat{\pi_{s}}$ denotes the model-implied proportion. Notation is simplified for illustrative purposes without sacrificing mathematical rigor. Asymptotically, the tail area of ^¯X2 can be approximated by a central chi-squared distribution with degrees of freedom S−1−2 , where −1 accounts for the constraint that the proportions must sum to 1, and −2 accounts for the location and scale of the summed score distribution (Li & Cai, 2018). The statistic can be used to detect the distributional assumption violations of the latent variable, with a lower statistic supporting the null hypothesis that the specified latent distribution is true. While this statistic suffices for the demonstration of the effectiveness of KDE, a correction factor can be applied if the statistic is used for formal hypothesis testing (see Li & Cai, 2018).

The resulting ^¯X2 statistics are 13.05 (*p*-value =0.042 ) for NM, 1.93 (*p*-value =0.926 ) for EHM, 7.55 (*p*-value =0.273 ) for DCM, and 6.26 (*p*-value =0.395 ) for KDE. The NM statistic rejects the null hypothesis under the type-I error level of 0.05, indicating that the normal distribution is an inadequate representation of the latent distribution. In contrast, EHM, DCM, and KDE substantially reduce ^¯X2 and yield higher *p*-values, showing that the estimated densities align well with the observed distribution.

Nevertheless, the unsmoothed EHM density may not be a realistic population distribution, potentially reflecting random noise, as suggested by Section 4. While both DCM and KDE produce practically acceptable population distributions, the slightly higher *p*-value for KDE may indicate a better fit between its estimated density and the true latent distribution.

## 6 Discussion

This article proposed a new approach to estimating the latent distribution of IRT, motivated by the recognition that violations of latent density assumptions can introduce bias into parameter estimates. The approach adopts the STE algorithm developed by Sheather and Jones (1991) to implement KDE, a widely recommended technique for general-purpose density estimation. By leveraging this method, the proposed approach enables accurate and efficient parameter estimation in IRT contexts. The article illustrated the statistical aspects of the method and evaluated it through a simulation study and an analysis of empirical data.

Although the use of the AMISE criterion to estimate latent density does not theoretically guarantee an increase in the likelihood—a common requirement in the EM algorithm literature (e.g., Minka, 1998; Neal & Hinton, 1998)—the convergence results from the simulation study demonstrated that the AMISE-based method yields robust EM convergence. Interestingly, although EHM and DCM are better aligned with the EM algorithm framework, KDE showed better convergence results than EHM and DCM; the EM convergence of EHM and DCM was often unsuccessful. This could imply that the AMISE criterion of KDE generally works in a way that increases the likelihood, even though it does not directly handle the likelihood.

The simulation study further demonstrated that the proposed method yields parameter estimates that are either superior to or on par with those obtained from existing methods, for both item and ability parameters. The model’s accuracy in parameter estimation can be attributed to its effectiveness in recovering the true latent distribution. The empirical analysis illustrated these findings: the estimated density of EHM could be unrealistic as a population distribution because of overfitting the data, and KDE provided a better match between the observed and model-implied summed score distributions than DCM for the CES-D8 dataset.

Meanwhile, alternative approaches can be utilized to address non-normality in the latent distribution, such as mixture IRT modeling (e.g., Sen & Cohen, 2019; von Davier & Rost, 2016). Whereas the latent density estimation in the context of this study assumes a single homogeneous population, mixture modeling posits that the data arise from a composite of latent subpopulations, thereby modeling non-normality using a mixture of the subpopulation distributions. When the research objective involves identifying latent classes, latent density estimates can serve as a diagnostic indicator of potential population heterogeneity.

From a practical standpoint, the proposed method also offers a significant computational advantage, as it does not require a model selection process. Existing methods to estimate the latent distribution of IRT typically require users to fit several models to determine the optimal degree of density smoothing through a model selection process. For example, 10 models are fitted to implement DCM as its hyperparameter *h* generally takes values of h=1,2,⋯,10 . In contrast, a single model fitting procedure suffices to employ KDE, thereby reducing a significant amount of computation time. This efficiency becomes even more valuable in the context of multidimensional IRT, where the number of quadrature points increases exponentially with the number of latent dimensions.

Future research could investigate the effectiveness of KDE under broader conditions, including applications to different IRT models, polytomous data, and multidimensional latent traits. When extended to multidimensional settings, the DPI algorithm can be used in place of the STE algorithm, which is currently limited to unidimensional estimation. Under these new scenarios, the comparative advantages of KDE demonstrated in this study should be re-evaluated within these new contexts.

Admittedly, considering that the performance of KDE was established on an average level through the simulation study, the estimated latent density from KDE might not always be the best solution for every given dataset. However, due to its stability in MML-EM convergence, accurate parameter estimation, and efficient computation time, KDE can be a good starting point to estimate the latent distribution of IRT when the normality assumption is in question.

## Appendix

**Table A1:** Simulation results of $RMSE(\hat{ICC})$ , $RMSE(\hat{a})$ , and $RMSE(\hat{b})$ Table A1 long description.

	Sample	No. of	$RMSE(\hat{ICC})$				$RMSE(\hat{a})$				$RMSE(\hat{b})$			
LD	size	items	NM	EHM	DCM	KDE	NM	EHM	DCM	KDE	NM	EHM	DCM	KDE
	500	8	0.032	0.052	0.032	0.032	0.273	0.683	0.274	0.267	0.117	0.233	0.117	0.118
	500	15	0.030	0.036	0.030	0.030	0.232	0.349	0.233	0.228	0.116	0.139	0.117	0.118
	500	30	0.029	0.032	0.029	0.029	0.211	0.260	0.211	0.209	0.116	0.123	0.117	0.117
	500	60	0.028	0.029	0.028	0.028	0.205	0.219	0.205	0.203	0.114	0.115	0.114	0.114
	1,000	8	0.023	0.039	0.023	0.023	0.189	0.366	0.190	0.185	0.084	0.166	0.084	0.084
	1,000	15	0.021	0.026	0.021	0.021	0.154	0.219	0.155	0.154	0.082	0.097	0.082	0.083
N	1,000	30	0.020	0.022	0.020	0.021	0.147	0.171	0.146	0.146	0.079	0.084	0.079	0.079
	1,000	60	0.020	0.020	0.020	0.020	0.143	0.151	0.143	0.142	0.078	0.079	0.078	0.078
	2,000	8	0.016	0.020	0.016	0.016	0.126	0.179	0.127	0.126	0.058	0.073	0.059	0.060
	2,000	15	0.015	0.019	0.015	0.015	0.110	0.149	0.110	0.111	0.059	0.072	0.059	0.060
	2,000	30	0.014	0.016	0.014	0.014	0.101	0.115	0.101	0.101	0.056	0.060	0.056	0.057
	2,000	60	0.014	0.015	0.014	0.014	0.098	0.103	0.098	0.098	0.057	0.057	0.057	0.057
	500	8	0.037	0.054	0.037	0.034	0.343	0.829	0.363	0.294	0.138	0.248	0.139	0.127
	500	15	0.033	0.036	0.032	0.030	0.275	0.359	0.278	0.234	0.139	0.145	0.129	0.126
	500	30	0.031	0.033	0.030	0.030	0.239	0.281	0.242	0.220	0.133	0.131	0.125	0.125
	500	60	0.029	0.029	0.029	0.029	0.214	0.235	0.222	0.209	0.122	0.120	0.119	0.119
	1,000	8	0.030	0.035	0.028	0.025	0.285	0.512	0.272	0.221	0.115	0.140	0.103	0.097
	1,000	15	0.026	0.026	0.023	0.022	0.217	0.244	0.194	0.165	0.109	0.104	0.091	0.091
S	1,000	30	0.023	0.023	0.021	0.021	0.181	0.188	0.167	0.151	0.096	0.091	0.085	0.085
	1,000	60	0.021	0.021	0.021	0.020	0.157	0.161	0.156	0.147	0.087	0.084	0.083	0.083
	2,000	8	0.026	0.026	0.020	0.019	0.248	0.235	0.191	0.168	0.102	0.116	0.076	0.077
	2,000	15	0.022	0.018	0.017	0.016	0.193	0.146	0.134	0.123	0.088	0.071	0.065	0.065
	2,000	30	0.019	0.016	0.015	0.015	0.152	0.125	0.119	0.110	0.077	0.064	0.062	0.061
	2,000	60	0.016	0.015	0.015	0.014	0.120	0.108	0.105	0.102	0.066	0.059	0.059	0.059
	500	8	0.034	0.038	0.034	0.032	0.335	0.414	0.335	0.289	0.116	0.138	0.116	0.111
	500	15	0.031	0.032	0.031	0.029	0.280	0.300	0.282	0.238	0.119	0.123	0.119	0.114
	500	30	0.028	0.029	0.029	0.027	0.228	0.240	0.242	0.205	0.110	0.109	0.110	0.108
	500	60	0.027	0.027	0.028	0.027	0.209	0.209	0.219	0.199	0.109	0.107	0.107	0.109
	1,000	8	0.026	0.029	0.026	0.023	0.255	0.383	0.257	0.208	0.089	0.110	0.089	0.081
	1,000	15	0.023	0.023	0.023	0.020	0.205	0.193	0.199	0.159	0.083	0.085	0.083	0.078
B	1,000	30	0.021	0.020	0.021	0.019	0.166	0.152	0.160	0.139	0.080	0.077	0.078	0.077
	1,000	60	0.019	0.019	0.019	0.019	0.148	0.141	0.145	0.136	0.076	0.074	0.074	0.075
	2,000	8	0.021	0.019	0.021	0.017	0.212	0.175	0.212	0.157	0.068	0.075	0.069	0.058
	2,000	15	0.018	0.016	0.016	0.014	0.166	0.128	0.133	0.111	0.063	0.059	0.059	0.055
	2,000	30	0.016	0.014	0.014	0.014	0.131	0.104	0.109	0.099	0.058	0.054	0.054	0.054
	2,000	60	0.014	0.013	0.013	0.013	0.105	0.095	0.096	0.093	0.055	0.053	0.053	0.053
	500	8	0.039	0.045	0.039	0.033	0.400	0.483	0.410	0.318	0.149	0.196	0.146	0.130
	500	15	0.034	0.034	0.033	0.029	0.314	0.337	0.295	0.235	0.142	0.130	0.126	0.122
	500	30	0.030	0.029	0.029	0.027	0.250	0.245	0.241	0.207	0.128	0.113	0.113	0.114
	500	60	0.028	0.027	0.028	0.027	0.216	0.221	0.223	0.204	0.116	0.111	0.111	0.112
	1,000	8	0.032	0.030	0.031	0.025	0.336	0.292	0.320	0.235	0.123	0.120	0.111	0.096
	1,000	15	0.027	0.022	0.023	0.021	0.263	0.198	0.201	0.166	0.115	0.088	0.088	0.087
SB	1,000	30	0.023	0.020	0.021	0.019	0.197	0.163	0.164	0.146	0.095	0.080	0.080	0.080
	1,000	60	0.020	0.019	0.019	0.019	0.161	0.147	0.148	0.140	0.083	0.076	0.076	0.077
	2,000	8	0.029	0.020	0.022	0.019	0.298	0.203	0.217	0.184	0.104	0.079	0.076	0.071
	2,000	15	0.023	0.017	0.018	0.015	0.231	0.146	0.155	0.120	0.092	0.065	0.066	0.060
	2,000	30	0.019	0.014	0.015	0.014	0.173	0.111	0.119	0.106	0.082	0.056	0.059	0.061
	2,000	60	0.015	0.014	0.014	0.014	0.123	0.101	0.104	0.098	0.064	0.055	0.056	0.056

*Note*: “LD,” “N,” “S,” “B,” and “SB” stand for latent distribution, normal, skewed, bimodal, and skewed bimodal, respectively.

**Table A2:** Simulation results of $ISE(\hat{g}(\theta ))$ and $RMSE(\hat{\theta })$ Table A2 long description.

	Sample	No. of	$ISE(\hat{g}(\theta ))$				$RMSE(\hat{\theta })$			
LD	size	items	NM	EHM	DCM	KDE	NM	EHM	DCM	KDE
	500	8	0.000	0.187	0.000	0.001	0.516	0.621	0.516	0.517
	500	15	0.000	0.127	0.000	0.001	0.406	0.442	0.406	0.407
	500	30	0.000	0.125	0.000	0.001	0.303	0.338	0.303	0.304
	500	60	0.000	0.117	0.000	0.001	0.223	0.246	0.223	0.224
	1,000	8	0.000	0.088	0.000	0.001	0.516	0.590	0.517	0.517
	1,000	15	0.000	0.060	0.000	0.001	0.403	0.423	0.404	0.404
N	1,000	30	0.000	0.051	0.000	0.001	0.301	0.320	0.301	0.302
	1,000	60	0.000	0.043	0.000	0.001	0.220	0.235	0.220	0.221
	2,000	8	0.000	0.037	0.000	0.000	0.513	0.521	0.513	0.513
	2,000	15	0.000	0.024	0.000	0.000	0.404	0.415	0.404	0.404
	2,000	30	0.000	0.020	0.000	0.000	0.298	0.308	0.298	0.299
	2,000	60	0.000	0.016	0.000	0.000	0.219	0.228	0.219	0.220
	500	8	0.017	0.202	0.018	0.010	0.532	0.643	0.532	0.524
	500	15	0.017	0.138	0.007	0.005	0.422	0.443	0.418	0.412
	500	30	0.017	0.125	0.003	0.003	0.315	0.337	0.311	0.308
	500	60	0.017	0.125	0.003	0.002	0.232	0.249	0.230	0.228
	1,000	8	0.017	0.113	0.010	0.008	0.532	0.568	0.527	0.523
	1,000	15	0.017	0.064	0.004	0.003	0.419	0.427	0.412	0.408
S	1,000	30	0.017	0.059	0.002	0.002	0.314	0.325	0.310	0.305
	1,000	60	0.017	0.048	0.002	0.001	0.228	0.237	0.227	0.224
	2,000	8	0.017	0.046	0.005	0.005	0.527	0.551	0.518	0.516
	2,000	15	0.017	0.028	0.002	0.002	0.419	0.413	0.410	0.406
	2,000	30	0.017	0.021	0.001	0.001	0.311	0.312	0.307	0.302
	2,000	60	0.017	0.018	0.001	0.001	0.228	0.230	0.226	0.223
	500	8	0.028	0.147	0.028	0.017	0.494	0.516	0.494	0.488
	500	15	0.028	0.108	0.026	0.009	0.389	0.403	0.389	0.379
	500	30	0.028	0.116	0.009	0.004	0.294	0.314	0.301	0.284
	500	60	0.028	0.109	0.004	0.003	0.215	0.222	0.219	0.210
	1,000	8	0.028	0.087	0.028	0.015	0.494	0.512	0.494	0.487
	1,000	15	0.028	0.047	0.012	0.007	0.389	0.391	0.387	0.377
B	1,000	30	0.028	0.038	0.003	0.003	0.293	0.298	0.296	0.283
	1,000	60	0.028	0.040	0.002	0.002	0.215	0.218	0.216	0.209
	2,000	8	0.028	0.034	0.023	0.013	0.490	0.494	0.488	0.482
	2,000	15	0.028	0.024	0.003	0.005	0.390	0.385	0.384	0.377
	2,000	30	0.028	0.015	0.001	0.002	0.292	0.289	0.291	0.282
	2,000	60	0.028	0.014	0.001	0.001	0.214	0.214	0.214	0.209
	500	8	0.075	0.168	0.074	0.049	0.499	0.560	0.499	0.484
	500	15	0.075	0.133	0.040	0.025	0.396	0.397	0.387	0.371
	500	30	0.075	0.136	0.016	0.010	0.298	0.301	0.293	0.278
	500	60	0.075	0.124	0.011	0.006	0.218	0.219	0.217	0.208
	1,000	8	0.075	0.092	0.065	0.043	0.499	0.507	0.496	0.482
	1,000	15	0.075	0.053	0.019	0.019	0.396	0.379	0.381	0.369
SB	1,000	30	0.075	0.045	0.011	0.008	0.299	0.290	0.289	0.277
	1,000	60	0.075	0.044	0.009	0.004	0.217	0.212	0.211	0.205
	2,000	8	0.075	0.041	0.032	0.036	0.495	0.484	0.478	0.475
	2,000	15	0.075	0.027	0.015	0.013	0.396	0.375	0.379	0.368
	2,000	30	0.075	0.020	0.010	0.005	0.296	0.282	0.284	0.274
	2,000	60	0.075	0.015	0.009	0.002	0.217	0.210	0.211	0.205

*Note*: “LD,” “N,” “S,” “B,” and “SB” stand for latent distribution, normal, skewed, bimodal, and skewed bimodal, respectively.

## Figure 1 Long description

Top left panel shows a density curve with bandwidth equals 0.4, which is jagged and closely follows the histogram bars below. Top right panel with bandwidth equals 0.7 shows a smoother curve, less jagged, with histogram bars below. Bottom left panel with bandwidth equals 1 shows an even smoother curve, approaching a normal distribution, with histogram bars below. Bottom right panel with bandwidth equals 1.3 shows the smoothest curve, nearly symmetric and bell-shaped, with histogram bars below. All panels have x axis labeled from negative 2 to 2 and y axis from 0 to 0.5. The main trend is that as bandwidth increases, the estimated density curve becomes smoother and less sensitive to local fluctuations.

Navigate back to Figure 1.

## Figure 2 Long description

Top left panel labeled Normal shows a symmetric bell-shaped curve for g of theta equals N open parenthesis 0 comma 1 squared close parenthesis, with x-axis theta from negative 4 to 4 and y-axis g of theta from 0 to 0.6. Top right panel labeled Bimodal shows two peaks, equation g of theta equals one half N open parenthesis negative 0.832 comma 0.555 squared close parenthesis plus one half N open parenthesis 0.832 comma 0.555 squared close parenthesis. Bottom left panel labeled Skewed shows a single peak shifted left, equation g of theta equals one half N open parenthesis negative 0.447 comma 0.566 squared close parenthesis plus one half N open parenthesis 0.447 comma 1.132 squared close parenthesis. Bottom right panel labeled Skewed bimodal shows two asymmetric peaks, equation g of theta equals one half N open parenthesis negative 0.832 comma 0.351 squared close parenthesis plus one half N open parenthesis 0.832 comma 0.702 squared close parenthesis. All panels have the same axis ranges.

Navigate back to Figure 2.

## Figure 3 Long description

There are three panels labeled a, b, and c from left to right. Panel a shows R M S E of I C C, panel b shows R M S E of a, and panel c shows R M S E of b. Each panel is divided into four rows for density types: Normal, Skewed, Bimodal, and Skewed Bimodal, and three columns for sample sizes: N equals 500, N equals 1000, N equals 2000. The x-axis in all subplots is number of items, ranging from 8 to 60. The y-axis varies by panel: panel a ranges from 0 to 0.05, panel b from 0 to 0.8, panel c from 0 to 0.25. Four methods are compared: N N (circle), E H M (triangle), P C M (square), and K D E (plus). Across all panels and density types, R M S E decreases as the number of items increases. The highest R M S E values are observed for smaller item counts and lower sample sizes, especially for the E H M method. The trend is consistent across density types, with Skewed and Skewed Bimodal densities showing slightly higher R M S E values. Method legend is shown at the bottom.

Navigate back to Figure 3.

## Figure 4 Long description

From the top left, panel a shows I S E of g hat theta for normal, skewed, bimodal, and skewed bimodal densities, each subdivided by N equals 500, 1000, and 2000. Lines represent N M, E H M, D C M, and K D E methods, with triangles, circles, squares, and plus signs. I S E decreases with more items, most notably for N M. Panel b, right, displays R M S E of theta hat for the same density and sample size structure. R M S E declines as item count rises, with all methods converging at higher item numbers. Legend at bottom right identifies method symbols. X axes are labeled number of items, Y axes are labeled I S E or R M S E.

Navigate back to Figure 4.

## Figure 5 Long description

The x axis is labeled theta, ranging from negative 4 to positive 4. The y axis is labeled latent density, ranging from 0.00 to 0.08. Four density curves are plotted: N M as a solid black line, E H M as a dotted black line, D C M as a dashed blue line, and K D E as a dash dotted red line. N M shows a symmetric bell-shaped curve centered at zero. E H M displays multiple peaks and troughs, with sharp increases near positive 2. D C M and K D E closely follow N M but with slight deviations, especially around the central peak. The legend at the top right links each line style to its method.

Navigate back to Figure 5.

## Table A1 Long description

The table presents simulation results for RMSE of estimated I C C, a, and b parameters. Columns from left to right are: latent distribution (L D), sample size, number of items, then for each parameter (I C C, a, b), four columns for methods N M, E H M, D C M, and K D E. L D codes are blank or one of N, S, B, S B, representing normal, skewed, bimodal, and skewed bimodal. Sample sizes are 500, 1,000, or 2,000. Number of items is 8, 15, 30, or 60. For each row, RMSE values are listed for each method and parameter. For example, the first row (blank L D, 500, 8) has RMSE for I C C: 0.032 (N M), 0.052 (E H M), 0.032 (D C M), 0.032 (K D E); for a: 0.273 (N M), 0.683 (E H M), 0.274 (D C M), 0.267 (K D E); for b: 0.117 (N M), 0.233 (E H M), 0.117 (D C M), 0.118 (K D E). As sample size and item count increase, RMSE values generally decrease across all methods and parameters. The table is divided into blocks by L D type, with each block showing the effect of increasing sample size and item count. The lowest RMSE values are observed for the largest sample sizes and item counts, especially for the N M and D C M methods. The footnote defines L D codes: L D is latent distribution, N is normal, S is skewed, B is bimodal, S B is skewed bimodal.

Navigate back to Table A1.

## Table A2 Long description

Column headers from left to right are: latent distribution (L D), sample size, number of items, I S E for N M, E H M, D C M, K D E, and R M S E for N M, E H M, D C M, K D E. L D types are blank, N, S, B, and S B, representing latent distribution, normal, skewed, bimodal, and skewed bimodal. For each L D, rows are grouped by sample size (500, 1,000, 2,000) and number of items (8, 15, 30, 60). For example, under L D blank, sample size 500, 8 items: I S E values are 0.000 (N M), 0.187 (E H M), 0.000 (D C M), 0.001 (K D E); R M S E values are 0.516 (N M), 0.621 (E H M), 0.516 (D C M), 0.517 (K D E). As sample size and item count increase, I S E and R M S E values generally decrease for all methods, with E H M showing higher I S E and R M S E than others. This trend is consistent across all L D types. For L D N, S, B, and S B, similar patterns are observed, with E H M consistently yielding higher error values. The lowest I S E and R M S E values are observed for higher sample sizes and item counts, especially for N M, D C M, and K D E methods. All data values are reported to three decimal places.

Navigate back to Table A2.
